# Performance of the BD Phoenix CPO detect assay for detection and classification of carbapenemase-producing organisms

**DOI:** 10.1007/s10096-020-04094-1

**Published:** 2020-11-27

**Authors:** Laura Berneking, Anna Both, Benjamin Berinson, Armin Hoffmann, Marc Lütgehetmann, Martin Aepfelbacher, Holger Rohde

**Affiliations:** Institut für Medizinische Mikrobiologie, Virologie und Hygiene, Universitätsklinikum Hamburg-Eppendorf, Martinistraße 52, D-20246 Hamburg, Germany

**Keywords:** Ambler class carbapenemase, BD Phoenix CPO detect, Carbapenemase-producing organisms, Carbapenemase-resistant, CPO, CRE, Enterobacterales

## Abstract

Increasing worldwide, prevalence of carbapenem-resistant gram-negative bacteria demands urgent a need for rapid detection and accurate identification of carbapenemases. The BD Phoenix CPO detect (PCD) assay possesses an in-built capacity for parallel susceptibility testing and detection of carbapenemases. Here, the ability of the assay to detect and classify carbapenemase production was tested in a collection of carbapenem-resistant *Enterobacterales* and non-fermentative gram-negative rods. The ability of the PCD assay to detect and classify carbapenemases was investigated in a collection of 194 clinical, carbapenem-resistant isolates (*Enterobacterales* [*n* = 65]; non-fermentative gram-negative rods [*n* = 129]). AST results were compared to MICS determined by gradient diffusion to determine accuracy of the PCD assay. The accuracy of the PCD assay to detect carbapenemases was compared to the results of molecular isolate characterization using a LDT multiplex carbapenemase PCR assay. All 194 isolates classified as carbapenem-resistant by reference susceptibility testing were also classified correctly as CRO by the PCD assay. Performance analysis of the PCD assay to detect carbapenemase production revealed an overall sensitivity of 98.29% and specificity of 17.95% for the detection of carbapenemase production. For the classification of carbapenemases classes A, B, and D, the PCD correctly classified 79.17% *Enterobacterales* and 67.16% non-fermentative gram-negative rods. The PCD assay is a reliable tool for the detection of carbapenem resistance and allows for parallel analysis of carbapenemase production. However, while sensitivity is high, low specificity in carbapenemase detection and erroneous classification demands mandatory confirmation by alternative methods, especially in non-fermentative gram-negative bacteria.

**Supplementary Information:**

The online version contains supplementary material available at 10.1007/s10096-020-04094-1.

## Introduction

Infections caused by carbapenem-resistant organisms (CRO) are emerging as a major challenge to public health [1, 2]. Over the past couple of years, a marked increase in the prevalence of CRE has been reported worldwide [3], and infections caused by CRO are associated with a significant mortality [4]. Of note, the incidence of invasive disease with CRE increases especially in high-risk populations, e.g., in patients treated in intensive care units and hematology wards [5]. Here, the majority of these infections are associated with increased length of stay and mortality rates ranging from 30 to 70% [6].

Gram-negative organisms essentially become carbapenem-resistant via two main routes, i.e., carbapenemase production or expression of cephalosporinases (AmpC) and/or extended spectrum β-lactamases (ESBL) in combination with cell wall permeability changes [7, 8]. Importantly, carbapenemases from molecular classes A, B, C, and D [9] are characterized by specific β-lactam hydrolytic profiles and susceptibility against inhibition by different β-lactamase inhibitors [10].

Therapeutic options for treatment of CRO-related infections are limited [11]. New promising therapy options for CRO are based on new β-lactams and β-lactam/β-lactamase inhibitor combinations (e.g., ceftazidime-avibactam, ceftolozane-tazobactam, meropenem-vaborbactam) [12]. These substances exhibit specific activity in CRO depending on the respective mechanism underlying carbapenem resistance, and in turn, determination of carbapenemase resistance mechanisms may already allow for informed decision making on optimal treatment [13].

Approaches to rapidly identify carbapenemase production in CRO include colorimetric assays (e.g., Carba NP), combined disk test (CDT), and the modified carbapenem inactivation method (mCIM). The long turnaround times (up to 24 h) of the two latter are a major limitation [14]. Faster identification by the available molecular multiplex PCR assays limited to the most frequent carbapenemase-encoding genes remain expensive [15, 16] and may have reduced sensitivity related to the possible presence of carbapenemases not included in the PCR panel. In that respect, a solution that combines antibiotic susceptibility testing with preliminary identification and classification of carbapenemases may represent an attractive alternative for rapid diagnostics of infections with CRO.

The BD Phoenix CPO detect (PCD) assay is the first automated test strategy that combines antimicrobial susceptibility testing with a built-in carbapenemase detection assay able to identify carbapenemase activity in clinical *Enterobacterales*, *Pseudomonas aeruginosa*, and *Acinetobacter baumannii*. In addition, the assay provides assignment to the respective four Ambler classes, thus potentially setting basis for rational choice of available antibiotics with dedicated CRO activity. The integration of the PCD assay into routine workflows may thus shorten the time to target antibiotic treatment. Here, we tested the performance of PCD in identifying carbapenemase production and assignment to Ambler classes in comparison to a multiplex carbapenemase PCR assay. Moreover, antibiotic susceptibility testing of meropenem, imipenem, and ceftazidime-avibactam was compared to minimal inhibitory concentrations (MICs) determined by gradient diffusion (Etest, bioMérieux; Liofilchem).

## Methods

### Bacterial isolates

CROs were collected at the University Medical Center Hamburg-Eppendorf, a 1600 bed maximum care hospital in northern Germany, between August 2015 and July 2018. Only first isolates of a patient grown from a clinical specimen (e.g., blood cultures, urine, and respiratory material) as well as screening materials (e.g., rectal swabs) were included. Species identification was performed using MALDI-TOF mass spectrometry (Bruker Biotyper, Bruker, Germany). Susceptibility of bacterial isolates was routinely tested on a Vitek 2 instrument (bioMérieux, Marcy-l’Etoile, France) using AST-N111 cards. Suspected CPO with resistance to meropenem or imipenem was confirmed by MIC determination using gradient diffusion assays (Etest, bioMérieux, Marcy-l’Etoile, France; Liofilchem, Supplemental Table 1). Results were interpreted according to EUCAST clinical breakpoints (version 10.0, 2020; *Enterobacterales*, imipenem/meropenem MICs > 4 g/L; *P. aeruginosa*/*A. baumannii*, imipenem MIC > 4 g/L/meropenem MIC > 8 g/L).

In total, 194 routine clinical isolates were included (*P. aeruginosa*, *n* = 108; *Acinetobacter baumannii*, *n* = 21; *Enterobacter* species, *n* = 11; *Klebsiella pneumoniae*, *n* = 39; *Klebsiella oxytoca*, *n* = 2; *Escherichia coli*, *n* = 9; *Serratia* species, *n* = 4). Per patient, only one isolate per species was included.

### Genotypic isolate characterization

All 194 isolates were tested for the presence of specific carbapenemase-encoding genes by a multiplex PCR [17], able to detect *bla*_KPC_, *bla*_IMP_, *bla*_VIM_, *bla*_NDM_, and *bla*_OXA-48-like_ and thus, the most common carbapenemase-encoding genes prevalent in Germany [18, 19]. Isolates negative in this assay were subsequently tested using a laboratory developed test (LDT) multiplex PCR assay for the presence of *bla*_GES_ (forward primer, 5′-TGGCTAAAGTCCTCTATG-3′, reverse primer 5′-CAACCCAATCTTTAGGAAA-3′, probe 5′-Fam-CGTCTCCCG/ZEN/TTTGGTTTCCG-3Iowa Black FQ-3′), *bla*_NMC-A_/_IMI_ (forward primer 5′-GTCACTTAATGTAAAACCAA-3′, reverse primer 5′-CTACCATTGAAATCTGTTTC-3′, probe 5′-Fam-AGCCATCTT/ZEN/GTTTAGCTCTTGTTTAGT-3Iowa Black FQ-3′, *bla*_BIC_ (forward primer 5′-GGAGAAACGTATCGACTATA-3′, reverse primer 5′-TCCAGAAGCAAATTTGTC-3′, probe (5′-Fam-CACCGTTGT/ZEN/CGCTGTACTGC-Iowa Black FQ-3′, and *bla*_SME_ (forward primer 5′-GGCTCAGGTATGACATTA-3′, reverse primer 5′-TCTCCAATAGAACGCATAA-3′, probe 5′-Fam-CTCAGGACC /ZEN/GCCAAGAAATCG-Iowa Black FQ-3′). *A. baumannii* isolates were additionally tested for the presence of genes encoding Oxa-type enzymes OXA-23-like and OXA-24-like according to a published protocol [20]. Table 1 provides an overview of carbapenemase-encoding genes detected in this study.

**Table 1:** Carbapenemase distribution in study isolates

	Class A		Class B			Class D					
Species	KPC	GES	VIM	NDM	IMP	Oxa-48 type	Oxa-23 type	Oxa-24 type	dual	PCR negative^a^	Total
*E. coli*			2	3		2				2	9
*K. pneumoniae*	2		1			27				9	39
*K. oxytoca*			2							0	2
*K. aerogenes*										3	3
*E. cloacae*			2	1		2				3	8
*S. marcescens*			1	1					2	0	4
*P. aeruginosa*		2	42		2	1			1	60	108
*A. baumannii*							17	1	1	2	21
Total	2	2	50	5	2	32	17	1	4	79	194

^a^PCR targeting *bla*_KPC_, *bla*_IMP_, *bla*_VIM_, *bla*_NDM_, *bla*_OXA-48-like_, *bla*_GES_, *bla*_NMC-A/IMI_, *bla*_BIC_, and *bla*_SME_. All PCR-negative isolates were phenotypically tested negative for carbapenemase production and thus, these isolates are assumed to express carbapenemase-independent carbapenem resistance

### Carbapenemase inactivation method

Isolates being carbapenemase-negative as detected by PCR were in addition tested for the presence of carbapenemase activity using the carbapenem inactivation method (CIM) [21].

### BD Phoenix CPO detect assay

The test was performed with the BD Phoenix NMIC-502 AST panel according to the manufacturers’ recommendations. Briefly, isolates were recovered from − 80 °C and inocula were prepared from overnight growth on Columbia agar plates + 5% sheep blood (BD Diagnostics Systems, Sparks, MD). NMIC-502 panels were inoculated using the Phoenix AP instrument and analyzed by the Phoenix M50 semi-automated AST system. Measured values were interpreted according to the algorithm-based Epicenter software provided by BD. The NMIC-502 panel consists of 27 wells with antimicrobial agents for antimicrobial susceptibility testing and further nine wells with assays to detect carbapenemases containing lactam agents with various combinations of ß-lactamase inhibitors. Isolates tested positive for carbapenemase activity might not be assigned to a specific Ambler class and therefore are interpreted as non-typeable. The BD Phoenix CPO detect panel provided MICs of imipenem and meropenem that were interpreted by a BD Phoenix algorithm and are included in the supplemental material (Table S1).

### Quality control

Quality controls were performed with *K. pneumoniae* ATCC700603 harboring SHV-18 extended spectrum β-lactamase as negative control and a laboratory *Escherichia coli* isolate BAA-1705 with a genetically determined Oxa-48-like carbapenemase as positive control.

### Statistical analysis

Sensitivity (true positives/[true positives + false negatives]) and specificity (true negatives/[true negatives + false positives]) with their respective 95% confidence intervals were calculated using the MedCal statistical software (MedCalc, 2018).

## Results

### Characterization of carbapenemase content in study isolates

By molecular characterization, carbapenemase-encoding genes were identified in 115/194 isolates (four class A carbapenemases [KPC, *n* = 2; GES, *n* = 2], 57 class B carbapenemases, [NDM, *n* = 5; IMP, *n* = 5; VIM, *n* = 51], 50 class D carbapenemases [Oxa-23, *n* = 17; Oxa-24, *n* = 1; Oxa-48, *n* = 32]). Four isolates produced more than one carbapenemase (*P. aeruginosa* (VIM and KPC), *n* = 1; *A. baumanii* (NDM and Oxa-24), *n* = 1; *Serratia marcescens* (Oxa-48 and VIM), *n* = 2). Carbapenem-resistant phenotypes in 79 carbapenemase-negative isolates (*P. aeruginosa*, *n* = 60; *K. pneumoniae*, *n* = 9; *E. coli*, *n* = 2; *Enterobacter* species, *n* = 6; *A. baumannii*, *n* = 2) were not further analyzed on a molecular level. However, these isolates all tested negative in the CIM assay and thus were assumed as isolates exhibiting a carbapenemase-independent carbapenem-resistant phenotype.

### Ability of the CPO assay to detect carbapenemases

All 194 isolates classified as CRO by reference susceptibility testing (Vitek II, gradient strip) were also found carbapenem-resistant by the PCD assay.

The PCD classified 178/194 CRO as carbapenemase-producing organisms. Compared to the reference method, the PCD called 113/115 (98.26%) carbapenemase-carrying isolates positive (true positives; *Enterobacterales*, *n* = 48, *P. aeruginosa*, *n* = 46, *A. baumannii*, *n* = 19). However, 65/79 (82.28 %) carbapenemase-negative isolates were also identified as CPOs by the PCD (false positives; *Enterobacterales*, *n* = 14, *P. aeruginosa*, *n* = 51). A total of 16/194 isolates (8.25%; *P. aeruginosa n* = 13; *Enterobacter* species, *n* = 3) were assigned as non-CPOs by the PCD, of which 14 were also carbapenemase-negative by the reference methods (true negative; *Enterobacterales*, *n* = 3, *P. aeruginosa*, *n* = 11). In two isolates, the PCD failed to detect a carbapenemase (false negative; *P. aeruginosa*, *n* = 2; Table 2). These findings result in an overall sensitivity of 98.29% (95% CI 93.96–99.79%) and specificity of 17.72% (95% CI 10.37–28.29%) for the detection of carbapenemase production by the PCD assay (Table 2). For non-fermenting organisms, the sensitivity and specificity were 97.01% (95% CI 89.68–99.48%) and 17.74% (95% CI 9.3–29.95%), respectively. For *Enterobacterales*, sensitivity and specificity were 100% (95% CI 90.77–100.00%) and 17.65% (95% CI 4.67–44.2%), respectively (Table 2).

**Table 2:** Performance of PCD assay for carbapenemase detection

PCD	PCR-positive (*n* = 115)	PCR-negative (*n* = 79)
Positive	113	65
*Enterobacterales*	48	14
Non-fermentative rods	65	51
Negative	2	14
*Enterobacterales*	0	3
Non-fermentative rods	2	11

### Ability of the CPO assay to differentiate carbapenemases

PCD-based assignment to a specific Ambler class was correct in 83/115 CPO, including *Enterobacterales* (38/48 [79.17%]) and non-fermentative gram-negative rods (45/67 [67.16%]) (Table S1).

A total of 9/10 misclassified *Enterobacterales* were not assigned to any specific Ambler class, while one *bla*_VIM_-carrying *S. marcescens* was misclassified as class D producing by the PCD assay. Two *P. aeruginosa* isolates (*bla*_Oxa-48_, *n* = 1; *bla*_VIM_, *n* = 1) were incorrectly assigned to class A, while no Ambler classification was provided for the additional 20 isolates (Table 3). Repeated testing of the same isolates again resulted in a wrong carbapenemase classification.

**Table 3:** Performance of PCD assay to classify carbapenemases

CPO type^a^	Class A	Class B	Class D	Unclassified positive	Negative
*Enterobacterales* (*n* = 48)					
Class A (*n* = 2)	1			1	
Class B (*n* = 13)		8	1	4	
Class D (*n* = 31)			27	4	
Dual (*n* = 2)		2		none	
Unclassified (*n* = 0)				10	
Non-fermenters (*n* = 67)					
Class A (*n* = 2)				2	2
Class B (*n* = 44)	1	30		13	
Class D (*n* = 19)	1		12	6	
Dual (*n* = 2)	1		1	none	
Unclassified (*n* = 0)				21	

^a^As classified by LDT multiplex PCR

Four strains with more than one carbapenemases-encoding gene (*P. aeruginosa* carrying *bla*_VIM_ and *bla*_KPC_, *n* = 1; *A. baumanii* carrying *bla*_NDM_ and *bla*_Oxa-24_, *n* = 1; *S. marcescens* carrying *bla*_VIM_ and *bla*_Oxa-48_, *n* = 2) were included in this study. The *P. aeruginosa* strain was identified as harboring a class A carbapenemase, the *A. baumannii* was identified as class D producers*,* while both *S. marcescens* dual producers were identified as producing a class B carbapenemase (Table S1).

## Discussion

Over the last years, a significant increase in CRO infections has been observed in many countries worldwide [4, 22]. The increasing clinical importance of CRO infections urgently demands methods for rapid identification and classification of carbapenemase in order to provide early optimized therapy. Indeed, early and accurate information on the presence and classification of a carbapenemase enables trained clinicians to make targeted use of new antibiotic substances, i.e., ceftazidime-avibactam or meropenem-vaborbactam [23]. In this respect, the PCD assay, marketed for testing antibiotic susceptibility and parallel identification of carbapenemase production, addresses a relevant clinical need. While carbapenemase detection and differentiation methods using immunological or molecular approaches provide faster results, the specific strength of an in-build phenotypic carbapenemase detection during routine susceptibility testing lies in the on-the-flight analysis character, providing evidence for carbapenemase production or exclusion of such activity for all isolates under routine investigation.

This study aimed to determine the usefulness of the PCD assay to detect carbapenemase production and to categorize respective enzymes according to the Ambler classification [24]. As previously described by others [8, 25, 26], the PCD assay provides accurate results for the identification of carbapenem resistance, with results available in less than 16 h. Moreover, the PCD assay proved able to identify carbapenemase production in our isolate collection with an overall high sensitivity (98.26%), but unexpectedly low specificity (17.72%). Five recent studies examined the analytical performance of the PCD test, using phenotypic carbapenemase detection assays (LDT Carba NP test, β-CARBA test, bioMérieux Rapidec Carba NP assay; [8, 25, 26]) or a PCR assay (BD MAX™ Check-Points CPO PCR assay; assay, in-house multiplex PCR; [27, 28]) as a reference. These studies found a comparable high overall sensitivity of 89.4–100%, but in contrast to the present study, a better specificity (68.6–87.1%) for the detection of carbapenemase activity in different collections of clinical *Enterobacterales* isolates [25, 26], partly including a small fraction of non-fermenters (18.03%, 17.09%, and 29.97%, respectively) [8, 27, 28].

The differences observed in the available studies regarding the specificity of PCD may be due to a number of different drivers. The studies differed grossly in overall sample size, with number of included isolates ranging from *n* = 95 to *n* = 294. In addition, the relative proportion of bacterial species substantially varied between studies, and in particular, the number of non-fermenters included exhibits large heterogeneity (*n* = 27 to *n* = 53; [8, 27, 28]), highlighting the potential impact of isolate selection on findings during assay evaluation studies. Especially, inclusion of carbapenem-resistant non-fermenters can potentially have a considerable influence on the test performance, given the lower pre-test probability for carbapenemase detection compared to *Enterobacterales*. Inherent problems in detecting carbapenemase production in non-fermenters relate to the expression of a broad spectrum of different β-lactam hydrolyzing enzymes (e.g., AmpC- or ESBL-type enzymes) readily leading to false positives in hydrolysis-based carbapenemase detection assays [21, 29, 30]. The present study included 108 *P. aeruginosa* and 21 *A. baumannii* strains, representing more than two-thirds of all tested isolates, which is in clear contrast to previously published studies. Inclusion of a large subset of challenging species may have made an important contribution to the overall unsatisfactory specificity of the PCD assay in the present study. In fact, compared to the reference methods used here, the PCD produced a high rate of false positive results in non-carbapenemase-producing *P. aeruginosa*, which accounted for 75.38% of all false positives

Discrepant false positive PCD results not necessarily must result from low specificity of the PCD, but certainly might also be related to false negative reference assay results. In fact, a limitation of our study is the restriction of the reference method to detect only the most common carbapenemase-encoding genes, including class A (KPC, GES, NMC-A/IMI, BIC, SME), class B (VIM, NDM, IMP), and class D (Oxa-48, Oxa-23, Oxa-24) carbapenemases [17, 31]. Although being the most prevalent and clinically relevant determinants of carbapenemase-mediated resistance in Germany [32–35], the presence of carbapenemases not covered by the PCR assay employed cannot be excluded. To compensate for the potential sensitivity limitation, all PCR-negative isolates were additionally tested using a phenotypic carbapenemase assay (i.e., CIM; [21]). While certainly, also this assay does not definitely exclude carbapenemase activity that might still be accessible for detection by the PCD. However, based on the combination of phenotypic and molecular methods applied to detect or exclude the presence of a carbapenemase in this isolate collection, it is plausible to assume that most of false positives are in fact a result of an overall low specificity of the PCD. Nevertheless, conclusive evidence certainly may only be obtained by whole genome sequencing.

Only two isolates tested were false negative for carbapenemase production, and both were found to carry *bla*_GES_. Indeed, GES-type carbapenemases [26] have previously been described as being difficult to detect by colorimetric methods [36]. The low number of false negatives is in line with previous studies, highlighting the strength of the PCD to serve as a tool to exclude presence of a carbapenemase in clinical isolates [27]. In fact, in a prospective study including 368/372 *Enterobacterales* isolates from various routine specimens were correctly identified as non-CPOs (own unpublished observation). To identify additional carbapenemase type-specific sensitivity problems of the PCD, a systematic analysis of isolates carrying rarer carbapenemase types will be necessary.

Given the clinical availability of novel β-lactamase inhibitors (i.e., avibactam, relebactam, vaborbactam) specific for defined subsets of carbapenemases, reliable information on the carbapenemase class may have immediate therapeutic implications. For example, detection of a class A carbapenemase could potentially allow start of ceftazidime-avibactam for therapy of a CRE, even if no standardized susceptibility testing is available [37]. To allow for such strategy, reliable, highly sensitive carbapenemase classification is mandatory. In the isolate collection analyzed here, the PCD assay correctly assigned a specific carbapenemase class in 79.17% of *Enterobacterales* and only 67.16% of non-fermenter isolates. These findings are in sharp contrast to results published recently, showing that 94.6% of isolates were correctly assigned to an Ambler class [27]. Others, however, reported similar low overall accuracy of 85.0%, 68.99%, and 78.95% for carbapenemase classification by the PCD [8, 25, 26].

In conclusion, data shown here demonstrate that the PCD assay is a reliable tool for the detection of CPOs with a high sensitivity, but low specificity. Implementation into workflows for routine analysis of clinical isolates thus demands additional confirmatory test to validate positive PCD results from the PCD analysis, especially for non-fermenter organisms. These should include assays allowing for definitive identification and typing of a carbapenemase. The accuracy of the in-build carbapenemase differentiation tool does not have the necessary accuracy to allow for its use in clinical decision making algorithms. The potential value of the PCD assay at present lies in its ability to exclude presence of carbapenemases, however, in a setting with low incidence of CRO, the usefulness in routine practice appears low.

## Supplementary Information

ESM 1 (XLSX 206 kb).

## References

[1] Grundmann H, Glasner C, Albiger B, Aanensen DM, Tomlinson CT, Andrasevic AT, Canton R, Carmeli Y, Friedrich AW, Giske CG, Glupczynski Y, Gniadkowski M, Livermore DM, Nordmann P, Poirel L, Rossolini GM, Seifert H, Vatopoulos A, Walsh T, Woodford N, Monnet DL, G. European Survey of Carbapenemase-Producing Enterobacteriaceae Working (2017). Occurrence of carbapenemase-producing Klebsiella pneumoniae and Escherichia coli in the European survey of carbapenemase-producing Enterobacteriaceae (EuSCAPE): a prospective, multinational study. Lancet Infect Dis.

[2] Ho J, Tambyah PA, Paterson DL (2010). Multiresistant Gram-negative infections: a global perspective. Curr Opin Infect Dis.

[3] Bonomo RA, Burd EM, Conly J, Limbago BM, Poirel L, Segre JA, Westblade LF (2018). Carbapenemase-producing organisms: a global scourge. Clin Infect Dis.

[4] Cassini A, Hogberg LD, Plachouras D, Quattrocchi A, Hoxha A, Simonsen GS, Colomb-Cotinat M, Kretzschmar ME, Devleesschauwer B, Cecchini M, Ouakrim DA, Oliveira TC, Struelens MJ, Suetens C, Monnet DL, A. M. R. C. G. Burden of (2019). Attributable deaths and disability-adjusted life-years caused by infections with antibiotic-resistant bacteria in the EU and the European Economic Area in 2015: a population-level modelling analysis. Lancet Infect Dis.

[5] Hughes J, Goldenberg SD, Tosas O, Edgeworth JD, Otter JA (2016). Recent emergence of carbapenem-resistant organisms in a low prevalence UK setting in London. J Infect Prev.

[6] Cai B, Echols R, Magee G, Arjona Ferreira JC, Morgan G, Ariyasu M, Sawada T, Nagata TD (2017). Prevalence of carbapenem-resistant gram-negative infections in the United States predominated by Acinetobacter baumannii and Pseudomonas aeruginosa. Open Forum Infect Dis.

[7] Codjoe FS, Donkor ES (2017). Carbapenem resistance: a review. Med Sci (Basel).

[8] Thomson G, Turner D, Brasso W, Kircher S, Guillet T, Thomson K (2017). High-stringency evaluation of the automated BD Phoenix CPO detect and Rapidec Carba NP tests for detection and classification of carbapenemases. J Clin Microbiol.

[9] Ambler RP (1980). The structure of beta-lactamases. Philos Trans R Soc Lond Ser B Biol Sci.

[10] Bush K, Bradford PA (2019). Interplay between beta-lactamases and new beta-lactamase inhibitors. Nat Rev Microbiol.

[11] Sheu CC, Chang YT, Lin SY, Chen YH, Hsueh PR (2019). Infections caused by carbapenem-resistant Enterobacteriaceae: an update on therapeutic options. Front Microbiol.

[12] Wong D, van Duin D (2017). Novel beta-lactamase inhibitors: unlocking their potential in therapy. Drugs.

[13] Tamma PD, Simner PJ (2018). Phenotypic detection of carbapenemase-producing organisms from clinical isolates. J Clin Microbiol.

[14] Tamma PD, Opene BN, Gluck A, Chambers KK, Carroll KC, Simner PJ (2017). Comparison of 11 phenotypic assays for accurate detection of carbapenemase-producing Enterobacteriaceae. J Clin Microbiol.

[15] Monteiro J, Widen RH, Pignatari AC, Kubasek C, Silbert S (2012). Rapid detection of carbapenemase genes by multiplex real-time PCR. J Antimicrob Chemother.

[16] Poirel L, Walsh TR, Cuvillier V, Nordmann P (2011). Multiplex PCR for detection of acquired carbapenemase genes. Diagn Microbiol Infect Dis.

[17] van der Zee A, Roorda L, Bosman G, Ossewaarde JM (2014). Screening rectal swabs for carbapenemase genes. J Clin Microbiol.

[18] Becker L, Kaase M, Pfeifer Y, Fuchs S, Reuss A, von Laer A, Sin MA, Korte-Berwanger M, Gatermann S, Werner G (2018). Genome-based analysis of carbapenemase-producing Klebsiella pneumoniae isolates from German hospital patients, 2008-2014. Antimicrob Resist Infect Control.

[19] Nordmann P, Poirel L (2019). Epidemiology and diagnostics of carbapenem resistance in gram-negative bacteria. Clin Infect Dis.

[20] Woodford N, Ellington MJ, Coelho JM, Turton JF, Ward ME, Brown S, Amyes SG, Livermore DM (2006). Multiplex PCR for genes encoding prevalent OXA carbapenemases in Acinetobacter spp. Int J Antimicrob Agents.

[21] van der Zwaluw K, de Haan A, Pluister GN, Bootsma HJ, de Neeling AJ, Schouls LM (2015). The carbapenem inactivation method (CIM), a simple and low-cost alternative for the Carba NP test to assess phenotypic carbapenemase activity in gram-negative rods. PLoS One.

[22] De Angelis G, Del Giacomo P, Posteraro B, Sanguinetti M, Tumbarello M (2020). Molecular mechanisms, epidemiology, and clinical importance of beta-lactam resistance in Enterobacteriaceae. Int J Mol Sci.

[23] Papp-Wallace KM (2019). The latest advances in beta-lactam/beta-lactamase inhibitor combinations for the treatment of Gram-negative bacterial infections. Expert Opin Pharmacother.

[24] Hall BG, Barlow M (2005). Revised Ambler classification of {beta}-lactamases. J Antimicrob Chemother.

[25] Ong CH, Ratnayake L, Ang MLT, Lin RTP, Chan DSG (2018). Diagnostic accuracy of BD Phoenix CPO detect for carbapenemase production in 190 Enterobacteriaceae isolates. J Clin Microbiol.

[26] Simon M, Gatermann S, Pfeifer Y, Reischl U, Gessner A, Jantsch J (2019). Evaluation of the automated BD Phoenix CPO Detect panel in combination with the beta-CARBA assay for detection and classification of carbapenemase-producing Enterobacterales. J Microbiol Methods.

[27] Croxatto A, Coste AT, Pillonel T, Bertelli C, Greub G, Prod'hom G (2020). Evaluation of the BD Phoenix CPO Detect Test for the detection of carbapenemase producers. Clin Microbiol Infect.

[28] Saad Albichr I, Anantharajah A, Dodemont M, Hallin M, Verroken A, Rodriguez-Villalobos H (2020). Evaluation of the automated BD Phoenix CPO detect test for detection and classification of carbapenemases in Gram negatives. Diagn Microbiol Infect Dis.

[29] Howard JC, Creighton J, Ikram R, Werno AM (2020). Comparison of the performance of three variations of the carbapenem inactivation method (CIM, modified CIM [mCIM] and in-house method (iCIM)) for the detection of carbapenemase-producing Enterobacterales and non-fermenters. J Glob Antimicrob Resist.

[30] Noel A, Huang TD, Berhin C, Hoebeke M, Bouchahrouf W, Yunus S, Bogaerts P, Glupczynski Y (2017). Comparative evaluation of four phenotypic tests for detection of carbapenemase-producing gram-negative bacteria. J Clin Microbiol.

[31] Thomson KS (2010). Extended-spectrum-beta-lactamase, AmpC, and carbapenemase issues. J Clin Microbiol.

[32] De Rosa A, Mutters NT, Mastroianni CM, Kaiser SJ, Gunther F (2019). Distribution of carbapenem resistance mechanisms in clinical isolates of XDR Pseudomonas aeruginosa. Eur J Clin Microbiol Infect Dis.

[33] Kaase M, Schimanski S, Schiller R, Beyreiss B, Thurmer A, Steinmann J, Kempf VA, Hess C, Sobottka I, Fenner I, Ziesing S, Burckhardt I, von Muller L, Hamprecht A, Tammer I, Wantia N, Becker K, Holzmann T, Furitsch M, Volmer G, Gatermann SG (2016). Multicentre investigation of carbapenemase-producing Escherichia coli and Klebsiella pneumoniae in German hospitals. Int J Med Microbiol.

[34] Kresken M, Korber-Irrgang B, Korte-Berwanger M, Pfennigwerth N, Gatermann SG, Seifert H, G. German Carbapenem Resistance Study (2020). Dissemination of carbapenem-resistant Pseudomonas aeruginosa isolates and their susceptibilities to ceftolozane-tazobactam in Germany. Int J Antimicrob Agents.

[35] Schafer E, Malecki M, Tellez-Castillo CJ, Pfennigwerth N, Marlinghaus L, Higgins PG, Mattner F, Wendel AF (2019). Molecular surveillance of carbapenemase-producing Pseudomonas aeruginosa at three medical centres in Cologne, Germany. Antimicrob Resist Infect Control.

[36] Bernabeu S, Dortet L, Naas T (2017). Evaluation of the beta-CARBA test, a colorimetric test for the rapid detection of carbapenemase activity in Gram-negative bacilli. J Antimicrob Chemother.

[37] Maurer FP, Christner M, Hentschke M, Rohde H (2017). Advances in rapid identification and susceptibility testing of bacteria in the clinical microbiology laboratory: implications for patient care and antimicrobial stewardship programs. Infect Dis Rep.

